# High-risk diagnosis combinations in patients undergoing interhospital transfer: a retrospective observational study

**DOI:** 10.1186/s12873-022-00742-1

**Published:** 2022-11-24

**Authors:** Andrew P. Reimer, Nicholas K. Schiltz, Siran M. Koroukian

**Affiliations:** 1 Frances Payne Bolton School of Nursing, Case Western Reserve University, 2120 Cornell Dr10900 Euclid Ave, Cleveland, OH 44106, 216-368-7570 USA; 2 Critical Care Transport, Cleveland Clinic, 9800 Euclid Ave, Cleveland, OH USA; 3 Population and Quantitative Health Sciences, School of Medicine, Case Western Reserve University, Cleveland, OH USA

**Keywords:** Electronic health records, Transportation of patients, Machine learning, Emergency helicopter, Helicopter ambulance

## Abstract

**Background:**

There is limited research on individual patient characteristics, alone or in combination, that contribute to the higher levels of mortality in post-transfer patients. The purpose of this work is to identify significant combinations of diagnoses that identify subgroups of post-interhospital transfer patients experiencing the highest levels of mortality.

**Methods:**

This was a retrospective cross-sectional study using structured electronic health record data from a regional health system between 2010–2017. We employed a machine learning approach, association rules mining using the Apriori algorithm to identify diagnosis combinations.

The study population includes all patients aged 21 and older that were transferred within our health system from a community hospital to one of three main receiving hospitals.

**Results:**

Overall, 8893 patients were included in the analysis. Patients experiencing mortality post-transfer were on average older (70.5 vs 62.6 years) and on average had more diagnoses in 5 of the 6 diagnostic subcategories. Within the diagnostic subcategories, most diagnoses were comorbidities and active medical problems, with hypertension, atrial fibrillation, and acute respiratory failure being the most common. Several combinations of diagnoses identified patients that exceeded 50% post-interhospital transfer mortality.

**Conclusions:**

Comorbid burden, in combination with active medical problems, were most predictive for those experiencing the highest rates of mortality. Further improving patient level prognostication can facilitate informed decision making between providers and patients to shift the paradigm from transferring all patients to higher level care to only transferring those who will benefit or desire continued care, and reduce futile transfers.

## Introduction

Approximately 1.6 million patients undergo interhospital transfer (IHT) each year in the United States [1]. Interhospital transfers are generally categorized as time sensitive, and non-time sensitive. Traditionally, time sensitive transfers (e.g., trauma, heart attack, stroke) require the highest and fastest level of transfer support. Transfer for these patients has been shown to be a life-saving measure, with reductions in mortality for trauma [2–8] and heart attack [9] patients, but has yielded conflicting results for stroke [10, 11] and minimally injured trauma patients [12–14].

However, a large proportion (~ 70%) of patients that undergo interhospital transfer consist of non-time sensitive patients, often with multiple diagnoses and comorbidities (e.g., sepsis complicated by respiratory and renal failure), who are being transferred from one hospital to another. For these non-time sensitive patients undergoing interhospital transfer, the limited research in this area indicates that they experience up to 3 times higher mortality [15, 16] while also experiencing double the length of stay and twice the cost compared to nontransferred patients [1].

There is limited research on individual patient characteristics, alone or in combination, that contribute to the higher levels of mortality in post-transfer patients. The long-term goal of this work is to use electronic health record data to develop clinical decision support systems (CDSS) to improve interhospital patient transfer.

The primary objective of this exploratory and descriptive work was to identify which patient conditions describe the highest levels of mortality post-IHT to help inform our future development of CDSS. To accomplish this, we included data from the entire episode of care, including the referring and receiving hospitalization, thus not eliminating potentially significant diagnoses that may not be known at the time of transfer. We ranked all combinations of diagnoses according to frequency and association with post-transport mortality.

## Methods

### Design

This is a retrospective cross-sectional exploratory descriptive study using existing electronic health records (EHR). The study was approved by the participating institutions IRBs (#14–1556 and #20,180,346). The data repository contains the EHR records for all patients within the health system that underwent IHT from one health system hospital to another.

### Study population

The study population includes all patients aged 21 and older that underwent critical care transfer within one Northeast Ohio health system from a community hospital to one of three main health system tertiary care receiving hospitals between 2010–2017.

### Outcomes

The main outcome of this study was in-hospital mortality defined as a binary variable.

### Health conditions

The main independent variables of interest were health conditions including primary and secondary diagnoses, acute medical problems, existing comorbidities, and past medical/surgical conditions. All diagnoses associated with the patient’s transport episode—associated hospital encounter leading to the transfer at the referring hospital and hospital encounter at the receiving hospital—were identified from clinical and administrative data sources. Diagnoses were identified through ICD-9-CM and ICD-10-CM billing codes and were then mapped to UMLS codes [17]. Then, to further leverage the individual diagnosis codes, we subcategorized each diagnosis code via the validated mapping algorithm described in more detail here, [18] that subcategorizes each diagnosis code into one of 6 categories: 1) primary or admitting diagnoses, 2) past medical, surgical or social history, 3) current problem list, 4) comorbidity, 5) discharge diagnoses, and 6) general diagnoses not mapped to one of the 5 identified categories. These subcategories provide additional detail beyond the diagnosis code by adding temporal information such as present on admission, and based off our previous work, increases the precision of using diagnosis codes in predictive model building that employ machine learning approaches. Once subcategorized, each condition was flagged as a binary present or not present (0/1) indicator for each patient. In rule-based analysis, these conditions formed the left-hand side of the rule, also known as the antecedent.

### Statistical analysis

The primary statistical analysis in this project was a rule-based machine learning method known as association rule mining. We used association rule mining (ARM) to identify the most common single, dyad, triad, and quadric combination of health conditions, and to identify which of these combinations were most highly associated with in-hospital mortality [19].

The ARM method creates “association rules” of the form X =  > Y, where X is one or more factors (in this study, health conditions) and Y is a single-item consequent (in our study, in-hospital mortality post-transfer) that X is associated with. ARM uses the *Apriori* algorithm to search all possible combinations of conditions within user defined constraints [20]. We limited the left-hand side to one, two, three and four-way combinations, and set a minimum left-hand support of 25 subjects. Support is the measure of prevalence for the rule or combination in the population, and confidence is the proportion of times that the rule is true. Lift is the ratio of the observed number (or percentage) of people with the combination divided by the expected number (or percentage) of people with that combination if each individual diagnosis was independent of one another [21]. We set a minimum improvement criterion of 10% to filter out rules that are redundant or offer little new information over more parsimonious rules [22]. Stated differently—the mortality (outcome) of a new rule has to be 10% higher than any of the subcombinations of the diagnoses included in the combination. For example, the overall mortality for the combination of acidosis and cardiac arrest is 72.6% which is at minimum 10% higher than either acidosis (25%) or cardiac arrest (59.3%) individually.

For each combination of diagnoses that met the minimum user defined constraints, we calculated the unadjusted odds ratio, and adjusted odds ratio using multivariable logistic regression with the combination as the main independent variable, in-hospital mortality post-transfer as the dependent, and controlling for age, sex and race. All analyses were conducted using R v.4.0.5 and RStudio v.1.3.959, along with the “arules” package v.1.7–3 [23].

## Results

Overall, 8893 patients were included in the analysis. Table 1 presents the patient characteristics. Overall, the mortality rate was 11.7% for patients undergoing critical care transfer. Patients experiencing increased mortality post-transfer were on average older (mean of 70.5 vs 62.6 years) and on average had more diagnoses in 5 of the 6 subcategories. A majority of patients were discharged home (52.6%) or to home with home care (9.3%).

Table 2 reports the most frequently occurring two, three and four-way diagnosis combinations. For the top two-way combinations, comorbidities and discharge diagnoses were the most prevalent diagnosis subcategories representing 10 of the 20 diagnoses. Comorbid essential hypertension and the general diagnosis of encounter for counseling were present in 3 of the top 4 combinations. For the top ten three-way combinations, comorbidities [14] and problem list [11] comprised 25 of the 30 diagnosis subcategories. The most common diagnosis was atrial fibrillation occurring in 4 of the top 10 combinations—3 occurrences subcategorized as an acute problem, and 1 occurrence as an existing comorbidity. Acute respiratory failure occurs in 4 of the 10 combinations subcategorized as an active medical problem. The following diagnoses appeared in 3 of 10 combinations: hypotension (active medical problem, congestive heart failure (comorbidity), and acidosis (comorbidity). For the top ten four-way combinations, comorbidities [19] and problem list [14] comprised 33 of the 40 diagnosis subcategories. The most common diagnosis was acute respiratory failure in 6 of the top 10 combinations, followed by acidosis and essential hypertension each appearing in 5 of the top 10 combinations. The top 7 two-way combinations were present in 50% or more of the sample and all co-occurred at least 4.2 times higher than expected in the study population. Similarly, all three-way combinations, and 9 of the four-way combinations each exhibited 43% or greater representation in the population with lifts 3.7 times higher. Note that these combinations are nonexclusive, so for instance, a person with seven comorbidities may appear in multiple two- or three-way groupings.

Table 3 lists the one, two, three and four-way combinations of diagnoses with the highest impact on mortality. A primary, or admitting diagnosis, of cardiac arrest was present in 1.6% of the study population and experienced a 59.3% mortality rate post-transfer with an aOR of mortality of 13.08 (95% confidence interval [CI] 9.21 – 15.57). Encounter for counseling was associated with the second highest aOR (13.01; 95% CI 7.75 – 21.83) for a single combination that occurred in 0.7% of the population. The combination of active medical problem of atelectasis and encounter for counseling was associated with an aOR of mortality 24.75 (95% CI 10.29 – 59.50). The top 5 two-way combinations that exhibited the highest aOR of mortality included at least one terminal diagnosis: encounter for counseling, cardiac arrest, or do not resuscitate. The bottom 5 two-way combinations that do not include a terminal diagnosis exhibit a significant drop in the aOR of mortality and are largely dominated by acute respiratory failure and congestive heart failure. The top three-way combination consists of active medical problems dyspnea, hypotension, and acute respiratory failure in 0.3% of the population with an aOR of mortality of 7.90 (95% CI 3.49—17.88). The top four-way combination consists of the comorbid conditions of hypertension, anemia and acidosis with an active medical problem of acute respiratory failure, exhibiting an aOR of mortality of 8.76 (85% CI 3.96 – 19.40).

## Discussion

A majority of the diagnoses contained in the three- and four-way combinations consisted of comorbidities. The influence of comorbidity on patient hospitalization and outcome is well documented. This work provides evidence of the influence of comorbidity in post-IHT mortality. Our previous work, and others, have shown that there is limited to no influence of transport factors such as how the patient was moved and interventions performed, impacting post-IHT outcome [24, 25]. In combination, these findings provide further support that patients may be on a trajectory that is minimally impacted by transfer, and thus has several important insights to consider regarding our approach to transfer decision making.

The diagnosis combinations yield insight into the role of comorbidity in patient outcomes post-transfer rather than admitting and discharge diagnoses. In fact, only the admitting diagnoses (pdx) of cardiac arrest and cerebral hemorrhage, history diagnoses (hx) acute renal failure with tubular necrosis and coagulopathy, and the discharge diagnoses (ddx) DNR, acute respiratory failure, kidney failure, and atrial fibrillation represented 13 of 100 total diagnoses. Further, there is a noticeable difference between the individual diagnoses subcategory composition when compared to the two-way and higher combinations, with 5 of 10 diagnoses from diagnosis subcategories discharge, history, and primary when compared to the two-way and greater combinations that are composed of a majority of comorbidities [38] and problem lists [35]. These differences highlight the significance of ARM in assessing combinations of diagnoses that yield a non-additive outcome, rather than the assessment of each covariate independently in regression models.

Unsurprisingly, those admitted with cardiac arrest experience high-mortality post-IHT. Transferring patients either during or after cardiac arrest continues to be a debated topic due to evidence of poor survival to hospital discharge [26–28]. Only within the setting of active myocardial infarction which can be reversed with timely cardiac catheterization, [29] is active transfer while in cardiac arrest non-controversial. While there remains limited evidence to guide transferring patients post-cardiac arrest, [30] our results provide initial evidence to spur further inquiry and clinical considerations. Two combinations of diagnoses, cardiac arrest in the presence of either a comorbidity of acidosis or active problem of hypotension, identified patients who experienced the highest rates of mortality post-IHT, and thus patients that may warrant further discussion with patient’s family or surrogates prior to making the decision to transfer. Alternatively, our data demonstrates that 40% of patients with a sending encounter diagnosis of cardiac arrest do survive hospitalization to discharge. Thus, further inquiry to identify phenotypes of patients at highest risk of mortality post-IHT and potentially not candidates for transfer, and alternatively, phenotypes of post-arrest patients that do benefit from IHT, and particularly the necessity of timely air transfer versus ground transfer, is needed.

The presence of the diagnosis encounter for counseling was an unexpected finding. Encounter for counseling originated from the administrative billing diagnosis source and was the generic code Z71.8. ICD-10-CM Z71 is the general code for person encounter health services for other counseling and medical advice and Z71.8 is a non-specific and non-billable code that requires additional specificity such as 0.89 that indicates advanced directives discussed with patient [31]. All but 2 of the encounters for counseling diagnoses were identified as end-of-life counseling, thus using the full code Z71.89 at time of code assignment would provide more insight that can be actionable earlier. Identifying patients who engage in end-of-life counseling post-transfer can provide an opportunity to intervene with patients or their surrogates prior to transfer. Combinations of diagnoses with high mortality rates or the combination of diagnoses that lead to high mortality post-IHT identified in this work provide insight into patients that may benefit from early engagement of palliative care prior to a transfer decision to establish goals of treatment and realistic expectations of outcome of care. Either in-house or teleconsulting with palliative care from the potential receiving institution can be added into the workflow. A contingent of patients may not decide to be transferred and continue care in their local hospital. Whereas, for those that do want to proceed with transfer, the mode and timing of transfer may then be assigned to ground rather than more expensive and limited resource air transfer as there is no longer an indication for time-sensitive transfer that will not confer morbidity or mortality benefit.

There were several limitations to this work. First, we used data from only one health system that may result in a selection bias for patients that undergo IHT. Second, we only used structured diagnosis codes from the EHR. While we included diagnoses from all structured sources of data, including reconciling clinical and administrative diagnosis codes going beyond just those codes entered and used in the EHR clinically, there is a possibility that additional diagnoses could be present in unstructured text sources. Lastly, we only used diagnosis codes for this analysis and did not include additional covariates beyond age, sex and race for the adjusted odds ratio calculations, thus there is potential for unmeasured confounding. However, the overall goal of this work to identify those at highest risk of mortality post-IHT was achieved.

Transferred patients experience higher mortality than patients that do not undergo interhospital transfer. The national rate of all-cause mortality for hospitalization is 2% [32] while all-cause mortality for interhospital transfers is 5.2% [1]. Our sample consisted of critical care transfers via both ground and air and admitted to all unit types exhibiting an overall mortality of 11.7%, similar to the 8.9% overall mortality reported for all transfers in another interhospital transfers cohort study [33]. Other studies have reported post-transfer mortality ranging from 16%—32% hospital mortality for transferred patients admitted to intensive care units [16, 34].

## Conclusion

In summary, we identified specific combinations of diagnoses with the highest mortality rates. Comorbid load, in combination with active medical problems, were the significant predictors for those experiencing the highest rates of mortality post-IHT. A majority of the diagnoses are not considered time sensitive which allows for the opportunity for further inquiry to identify interventions to either avoid IHT or help make patient centered decisions and triage to the most appropriate mode and level of transport. The diagnosis combinations identified in this work provide a starting point for future research aimed at developing richer phenotypes of patients that can be used to facilitate patient-centered care. Precise patient level prognostication will facilitate informed decision making between providers, and patients and their families to shift the paradigm from transferring all patients to higher level care to only transferring those patients that will benefit or for patients that desire continued care.

**Table 1:** Patient demographics

**Patient Demographics**	*N* = 8,893	Alive	Dead
**Age** mean (± std dev)	63.5 (14.3)	62.6 (16.0)	70.5 (14.0)
range	21 – 90^a^	21—90	21—90
**Sex** n (%)			
Male	4,755 (53)	4,247 (89)	508 (11)
Female	4,138 (47)	3,605 (87)	533 (13)
**Race** n (%)			
White	6,531 (73)	5,773 (88)	758 (12)
Black	1,973 (22)	1,734 (88)	239 (12)
Other	389 (4)	342 (88)	44 (12)
**Diagnosis Subcategories** mean (± std dev)			
Primary Diagnosis		3 (2)	2 (1)
Problem List		5 (5)	6 (5)
Diagnosis		8 (8)	9 (8)
Comorbidity		6 (6)	8 (6)
History		2 (3)	3 (3)
Discharge Diagnosis		1 (5)	2 (6)
**Discharge Disposition** n (%)			
Home	4,683 (52.6)		
Died	1,041 (11.7)		
Home care	828 (9.3)		
Nursing facility	1,566 (17.6)		
Other facility	677 (7.6)		
Other	98 (1.1)		

*Std dev*   Standard deviation

^a^age limited to 90 for those aged 90 or greater due to deidentification

**Table 2:** Two-Way and Three-Way Comorbidity Combinations with the Highest Prevalence

Diagnosis 1	Diagnosis 2	Diagnosis 3	Diagnosis 4	Lift	%
**Two-way combinations**					
pl_Atelectasis	dx_Encounter.for.counseling			6.54	76.7
cm_Essential.Hypertension	ddx_DNR			6.33	74.2
cm_Acidosis	pdx_Cardiac.Arrest			6.20	72.6
cm_Essential.Hypertension	dx_Encounter.for.counseling			6.19	68.0
pl_Hypotension	pdx_Cardiac.Arrest			5.80	65.9
pl_Acute.respiratory.failure	pdx_Cerebral.Hemorrhage			4.70	55.1
cm_Congestive.heart.failure	hx_ARF.with.tubular.necrosis			4.27	50.0
cm_Altered.mental.status	hx_Atherosclerosis.without.angina			4.10	48.0
ddx_Kidney.Failure.Acute	ddx_Atrial.Fibrillation			4.10	48.0
ddx_Coronary.atherosclerosis.of.native	ddx_Acute.respiratory.failure			4.03	47.2
**Three-way combinations**					
cm_Essential.Hypertension	dx_Abmoral.EKG	dx_Encounter.for.counseling		6.57	77.0
cm_Acidosis	pl_Atrial.Fibrillation	pl_Hypotension		4.27	50.0
pl_Dyspnea	pl_Hypotension	pl_Acute.respiratory.failure		4.10	48.0
cm_Congestive.heart.failure	dx_Abmoral.EKG	pl_Atrial.Fibrillation		4.13	48.4
pl_Kidney.Failure.Acute	cm_Acidosis	cm_Chronic.atrial.fibrillation		4.11	48.2
cm_Congestive.heart.failure	pl_Acute.respiratory.failure	dx_abnormal.blood.chemistry		3.96	46.4
cm_Anemia	cm_Acidosis	dx_Long.term.use.of..medications		3.92	46.0
cm_Essential.Hypertension	pl_Acute.respiratory.failure	cm_Chronic.Kidney.Diseases		3.90	45.7
cm_Congestive.heart.failure	pl_Atrial.Fibrillation	cm_Altered.mental.status		3.79	44.4
cm_Essential.Hypertension	pl_Acute.respiratory.failure	cm_Altered.mental.status		3.75	43.9
**Four-way combinations**					
cm_Essential.Hypertension	cm_Anemia	cm_Acidosis	pl_Acute.respiratory.failure	4.44	52.0
pl_Kidney.Failure.Acute	cm_Anemia	cm_Acidosis	pl_Acute.respiratory.failure	4.27	50.0
pl_Kidney.Failure.Acute	cm_Acidosis	pl_Acute.respiratory.failure	cm_Chronic.Kidney.Diseases	4.12	48.3
pl_Dyspnea	pl_Kidney.Failure.Acute	cm_Acidosis	cm_Chronic.kidney.disease.stage.3.moderate	3.96	46.4
pl_Kidney.Failure.Acute	cm_Congestive.heart.failure	cm_Acidosis	cm_Chronic.Kidney.Diseases	3.96	46.4
cm_Essential.Hypertension	dx_Encounter.due.to.tobacco	pl_Acute.respiratory.failure	ddx_Coronary.atherosclerosis.of.native	3.94	46.2
cm_Essential.Hypertension	dx_Abmoral.EKG	pl_Hypotension	dx_ECG.abnormalities.non.specific	3.94	46.2
cm_Essential.Hypertension	dx_Encounter.due.to.tobacco	pl_Acute.respiratory.failure	hx_Coronary.atherosclerosis.of.native	3.79	44.4
pl_Kidney.Failure.Acute	cm_Congestive.heart.failure	pl_Acute.respiratory.failure	cm_Chronic.kidney.disease.stage.3..moderate.	3.70	43.3
cm_Essential.Hypertension	dx_Abmoral.EKG	cm_Acidosis	pl_Hypotension	3.47	40.6

Lift is the ratio of the observed number (or percentage) of people with the combination divided by the expected number (or percentage) of people with that combination if each individual diagnosis was independent of one another

The order of the conditions (diagnosis 1, diagnosis 2, or diagnosis 3) in the combination does not matter

*pl* Problem list, *dx* Unassigned diagnosis, *cm* Comorbidity, *pdx* Primary or admitting diagnosis, *hx* History diagnosis *ddx* Discharge diagnosis

**Table 3:** One-Way, Two-Way, and Three-Way Diagnosis Combinations with the Highest Impact on Mortality

**Combination of comorbidities**	**% with combination, coverage**	**% died in-hospital, confidence**	**Odds** **ratio**	**Adjusted odds ratio**	**95% confidence interval**	
**Individual diagnoses**						
pdx_Cardiac.Arrest	1.6	59.3	11.89	13.09	9.21-18.57	
dx_Encounter.for.counseling	0.7	64.7	14.39	13.01	7.75-21.83	
ddx_DNR	0.4	65.9	14.97	12.12	6.35-23.14	
pl_Acute.respiratory.failure	9.6	29.4	3.83	4.00	3.37-4.73	
dx_Other.ascites	1.1	28.0	2.98	3.56	2.26-5.60	
ddx_Acute.respiratory.failure	1.2	33.3	3.87	3.39	2.23-5.14	
cm_Acidosis	12.6	25.0	3.07	3.37	2.87-3.95	
dx_Hypothermia.due.to.exposure	0.5	30.4	3.33	3.26	1.69-6.26	
hx_ARF.with.tubular.necrosis	0.9	24.4	2.46	2.65	1.57-4.45	
hx_Coagulopathy	1.3	29.1	3.16	2.54	1.68-3.84	
**Two-way combination**						
pl_Atelectasis & dx_Encounter.for.counseling	0.3	76.7	25.32	24.75	10.29-59.50	
cm_Acidosis & pdx_Cardiac.Arrest	0.5	72.6	20.63	21.97	11.62-41.52	
pl_Hypotension & pdx_Cardiac.Arrest	0.2	68.0	16.27	20.12	8.53-47.47	
cm_Essential.Hypertension & ddx_DNR	0.3	74.2	22.15	18.22	7.95-41.72	
cm_Essential.Hypertension & dx_Encounter.for.counseling	0.4	72.5	20.42	16.37	8.02-33.36	
pl_Acute.respiratory.failure & pdx_Cerebral.Hemorrhage	0.5	55.1	9.47	8.37	4.68-14.96	
cm_Congestive.heart.failure & hx_ARF.with.tubular.necrosis	0.3	50.0	7.63	6.72	3.14-14.35	
pl_Atelectasis & ddx_Acute.respiratory.failure	0.3	42.3	5.57	5.71	2.54-12.79	
pl_Acute.respiratory.failure & dx_Encounter.due.to.CABG	0.4	44.4	6.11	5.55	2.82-10.91	
ddx_Kidney.Failure.Acute & ddx_Atrial.Fibrillation	0.2	48.0	7.03	5.55	2.47-12.43	
**Three-way combination**						
pl_Dyspnea & pl_Hypotension & pl_Acute.respiratory.failure	0.3	48.0	7.03	7.90	3.49-17.88	
cm_Acidosis & pl_Atrial.Fibrillation & pl_Hypotension	0.4	50.0	7.65	6.91	3.46-13.76	
cm_Essential.Hypertension & pl_Hypotension & dx_ECG.abnormalities.non.specific	0.3	42.9	5.71	6.32	2.93-13.60	
cm_Anemia & cm_Acidosis & dx_Long.term.use.of..medications	0.4	46.0	6.50	6.00	3.07-11.70	
pl_Kidney.Failure.Acute & cm_Acidosis & cm_Chronic.atrial.fibrillation	0.6	48.2	7.07	5.94	2.72-12.95	
cm_Congestive.heart.failure & cm_Acidosis & dx_Long.term.use.of..medications	0.6	43.2	5.82	5.92	2.98-11.73	
cm_Congestive.heart.failure & pl_Acute.respiratory.failure & dx_abnormal.blood.chemistry	0.3	46.4	6.60	5.58	2.57-12.10	
cm_Essential.Hypertension & pl_Acute.respiratory.failure & cm_Chronic.Kidney.Diseases	0.4	45.7	6.43	5.57	2.81-11.00	
cm_Anemia & cm_Acidosis & pl_Acute.respiratory.failure	0.7	40.0	5.13	5.32	3.19-8.87	
cm_Essential.Hypertension & pl_Hypotension & pl_Acute.respiratory.failure	0.6	41.1	5.35	5.26	3.03-9.13	
**Four-way combination**						
cm_Essential.Hypertension & cm_Anemia & cm_Acidosis & pl_Acute.respiratory.failure	0.2	52.0	8.26	8.76	3.96-19.40	
pl_Kidney.Failure.Acute & cm_Anemia & cm_Acidosis & pl_Acute.respiratory.failure	0.3	50.0	7.63	7.75	3.65-16.47	
pl_Kidney.Failure.Acute & cm_Acidosis & pl_Acute.respiratory.failure & cm_Chronic.Kidney.Diseases	0.3	48.3	7.12	7.53	3.56-15.91	
cm_Essential.Hypertension & dx_Abmoral.EKG & pl_Hypotension & dx_ECG.abnormalities.non.specific	0.2	46.2	6.53	7.02	3.19-15.48	
pl_Kidney.Failure.Acute & cm_Congestive.heart.failure & cm_Acidosis & cm_Chronic.Kidney.Diseases	0.3	46.4	6.61	6.49	3.02-13.95	
pl_Dyspnea & pl_Kidney.Failure.Acute & cm_Acidosis & cm_Chronic.kidney.disease.stage.3.moderate	0.3	46.4	6.61	5.43	2.54-11.61	
pl_Dyspnea & pl_Kidney.Failure.Acute & cm_Acidosis & cm_Chronic.Kidney.Diseases	0.3	39.3	4.92	5.28	2.40-11.61	
pl_Kidney.Failure.Acute & cm_Acidosis & pl_Atrial.Fibrillation & pl_Acute.respiratory.failure	0.2	40.0	5.07	5.20	2.29-11.80	
cm_Essential.Hypertension & dx_Abmoral.EKG & cm_Acidosis & pl_Hypotension	0.3	35.5	5.21	5.19	2.51-10.74	
cm_Essential.Hypertension & dx_Encounter.due.to.tobacco & pl_Atrial.Fibrillation & pl_Acute.respiratory.failure	0.4	39.3	6.56	4.92	2.59-9.38	

*pl* Problem list, *dx* Unassigned diagnosis, *cm* Comorbidity, *pdx* Primary or admitting diagnosis, *hx* History diagnosis, *ddx* Discharge diagnosis

## Data Availability

The datasets used and analysed during the current study are available from the corresponding author on reasonable request.
